# The effectiveness of minimally-invasive corticotomy-assisted orthodontic treatment of palatally impacted canines compared to the traditional traction method in terms of treatment duration, velocity of traction movement and the associated dentoalveolar changes: A randomized controlled trial

**DOI:** 10.12688/f1000research.135338.1

**Published:** 2023-06-19

**Authors:** Mahran R. Mousa, Mohammad Younis Hajeer, Ahmad S. Burhan, Omar Heshmeh, Mohammad Khursheed Alam

**Affiliations:** 1 Department of Orthodontics, University of Damascus Faculty of Dentistry, Damascus, Damascus, Syria; 2 Department of Oral and Maxillofacial Surgery, University of Damascus Faculty of Dentistry, Damascus, Damascus, Syria; 3 Orthodontic Division, Department of Preventive Dentistry, College of Dentistry, Jouf University, Sakaka, 72345, Saudi Arabia

**Keywords:** Upper impacted canine, palatally impacted canine, unerupted canine, forced eruption, orthodontic, treatment, acceleration, corticotomy, piezosurgery, CBCT imaging

## Abstract

**Objective:** To evaluate the effectiveness of a minimally-invasive corticotomy-assisted treatment of palatally impacted canines (PICs) compared with the traditional method by evaluating treatment time, the velocity of movement, and the associated dentoalveolar changes.

**Materials and methods:** Forty-six patients with palatally or mid-alveolar upper impacted canines were recruited and distributed into two groups: the corticotomy-assisted traction group (CAT group, mean age: 20.39±2.27 years) and the traditional treatment group (TT group, mean age: 20.26±2.17 years). The closed surgical approach was used in both study groups. The velocity of traction movement, traction duration and overall treatment duration were evaluated clinically. In addition, the bone support ratios and the amount of root resorption were assessed on cone-beam computed tomography (CBCT) images.

**Results:** At the end of treatment, significant differences were found between the two groups regarding the velocity of traction movement, traction time, and overall treatment time (P<0.05). The mean velocity of traction movement in the CAT group was greater than the TT group ( *x*_velocity_=1.15±0.35 mm/month; 0.70±0.33 mm/month, P=0.027, respectively). The duration of the active traction and the overall orthodontic treatment in the CAT group were significantly shorter than the TT group by 36% and 29%, respectively. The mean bone support ratios of the aligned canines did not differ significantly between the two groups (88% vs. 89% in the CAT and TT groups, respectively). No significant differences were found between the two groups regarding the mean amount of root resorption on the adjacent laterals ( *x*_resorption_ = 1.30±1.18 mm; 1.22±1.02 mm, P=0.612, in CAT and TT groups, respectively).

**Conclusions:** The traction movement velocity of the palatally impacted canines can be increased using minimally-invasive corticotomy-assisted orthodontic treatment. The side effects of the acceleration procedure were minimal and almost similar to those of the traditional technique.

## Introduction

There are several approaches to treat the impacted maxillary canines, including no treatment, ^1^ interceptive treatment, ^2^ extraction, ^3^ auto transplantation, ^4^ and orthodontic traction after surgical exposure. ^5^ According to clinical experience, the surgical/orthodontic approach is a highly successful treatment method, especially in cooperating patients. ^6^ The time required for orthodontic traction of impacted canines is a particularly troubling clinical problem because it prolongs the orthodontic treatment duration. ^7^

The duration of the surgical/orthodontic treatment ranged between 18-30 months. ^8^^,^^9^ This is related to many factors, including: the patients’ age, the severity of dental crowding, the initial inclination of the impacted canines, the bucco-lingual impacted canine’s position, the distance from the occlusal plan and the periodontal health. ^10^

Stewart *et al*., ^11^ in a retrospective study, identified the factors that could affect the duration of treatment in patients with palatally impacted maxillary canines by age, sex, severity of impaction, amount of crowding, and unilateral or bilateral impaction. They found that the duration of treatment was 25.8 months for unilateral impaction cases, and in bilateral cases, the duration of treatment was 32.3 months. They showed that if the impacted canine was less than 14 mm away from the occlusal plan, the average treatment duration was 23.8 months, while the average treatment duration in cases of impaction of more than 14 mm was about 31.1 months. ^11^

According to the comparative prospective study by Parkin *et al.,* ^12^ the effective traction period is defined as the period from the first application of the orthodontic traction force until the possibility of applying the first rectangular archwire. While the total treatment duration is defined as the period between the bonding of orthodontic brackets and to the removal of the orthodontic appliance. They demonstrated that focusing on the duration of active treatment is more important than the total duration of treatment that focuses on correcting other factors associated with malocclusion. ^12^

Becker and Chaushu ^13^ in a comparative retrospective study compared a group of adult patients (mean age 28.8 years) with a group of young patients (mean age of 13.7 years) who had palatally impacted canines. They found a significant increase in the treatment duration in the adult patients group (22.3±10.7 months and 19.6±9.1 months, in the adult and young patient groups, respectively) and a decrease in the success rate of treatment. On the other hand, Zuccati *et al.* ^14^ concluded that patients with palatally impacted canines over the age of 25 years required an average of 30 more visits than those who were under 25 years of age (the mean number of visits was about 40).

The type of surgical exposure technique may affect the treatment duration of impacted canines, ^14^ with conflicting evidence available. ^12^^,^^15^^–^^17^ The results of a systematic review by Mousa *et al.* ^18^ showed that the duration of orthodontic treatment of palatally impacted canines could be reduced when the open traction technique was combined with either superelastic wires or elastic traction elements. This review also concluded that the use of mini screws as a direct anchorage means can reduce the treatment duration and that the use of acceleration techniques (surgical and non-surgical) can increase the velocity of traction movement. ^18^

The use of various techniques to accelerate tooth movement during orthodontic treatment of different types of malocclusion, such as retraction of upper canines, ^19^^–^^21^ decrowding of lower anterior teeth, ^22^^–^^24^ retraction of maxillary anterior teeth, ^25^^,^^26^ the intrusion of posterior teeth, ^27^ and relieving upper dental crowding, ^28^ has recently been gaining more and more attention. In contrast, a few articles have been published on accelerating the traction movement of the maxillary impacted canines either by surgical or non-surgical accelerating methods. ^29^^–^^31^

The preliminary split-mouth design study conducted by Fischer ^29^ concluded that there was an acceleration in the traction movement of the palatally impacted canines by 28% to 33% when using the corticotomy-assisted technique compared to the conventional traction method. However, the results of this study remain not fully reliable due to the small sample size (only six patients).

Ferguson *et al.,* ^30^ in their retrospective cohort study, evaluated the effectiveness of the ostectomy-decortication technique in the forced-eruption duration of palatally impacted canines. They found that the impacted canines moved about 3.2 times faster in the surgical intervention group than in the conventional traction group, but the acceleration technique used in this study was invasive and involved the removal of a large amount of bone, which in turn could have affected the level of bone support around the aligned canines and their adjacent teeth.

However, the potential effects of such surgical interventions on the root resorption and bone support of the canines and their adjacent teeth have not been evaluated in these previous two studies. Finally, Yussif *et al.,* ^31^ in a randomized controlled trial, used a non-surgical acceleration method that involved repeated local injections of vitamin C into the impaction area during orthodontic traction of the palatally impacted canines. They found that the rate of movement of the impacted canines in the acceleration group was 2-2.5 mm/month compared to 0.5-1.5 mm/month in the control group. Despite the effectiveness of this method in accelerating the withdrawal movement, the acceleration procedure used may be associated with significant levels of discomfort and patient’s dissatisfaction. The main aim of the current study was to evaluate the effectiveness of corticotomy, applied at the exposure operation time and in a flapless manner two months post-operatively, on accelerating canine withdrawal in cases with unilateral maxillary canine impactions. The secondary objectives were to evaluate the ratios of bone support of the aligned canine, the adjacent lateral incisor and first premolar, as well as the root resorption status of the adjacent lateral incisor.

## Methods

### Study design and registration

The current study was designed as a randomized, controlled, two-parallel group clinical trial to investigate the efficacy of the corticotomy technique accompanying surgical exposure enhanced by a subsequent flapless corticotomy in accelerating traction movement of the palatally impacted canines (PICs) and to evaluate the associated dentoalveolar changes compared with traditional traction method **.** The study sample was compiled by reviewing the lists of patients recorded in the archives of the Orthodontics Department at the Faculty of Dentistry, Damascus University. The patients who were referred to the Orthodontic Department for treatment of impacted upper canines diagnosed in other departments were also examined. The approval of the Local Research Ethics Committee of Damascus University was obtaining before conducting this study (UDDS-499-01022020/SRC-2195). This study was registered at ClinicalTrials.gov and was funded by the Postgraduate Research Budget of Damascus University (Ref no: 83005751015DEN).

### Calculating the sample size

The desired sample size was established using Minitab ^®^ version 17 software (Minitab Inc., State College, Pennsylvania, USA). A proprietary free alternative is G*Power 3.1.9.7 program (the Heinrich-Heine University, Dusseldorf, Germany). It was believed that 0.25 mm/month would be the least clinically meaningful difference requiring detection in the impacted canine’s movement velocity between the two study groups. The standard deviation of this variable, based on a comparable prior study, was 0.278. ^29^ An independent-samples t-test (significance level 5%, power 80%) revealed that each group needed a minimum of 21 patients. To account for the possibility of patient withdrawal, each group’s patient count was increased to 23.

### Recruitment of patients and eligibility standards

**Figure 1.**
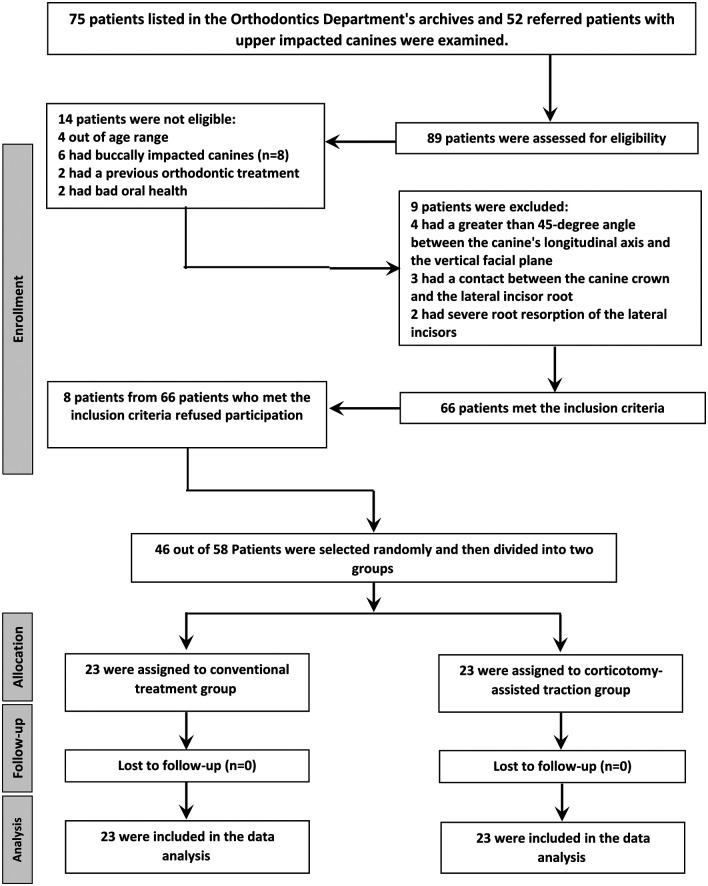
CONSORT flow diagram of patients' recruitment, follow-up, and entry to data analysis.

Along with contacting and recalling 75 individuals listed in the Orthodontics Department’s archives, 52 referred patients with upper impacted canines were also examined. It was found that 66 of them satisfied the inclusion requirements. Fifty-eight patients were accepted to participate in the study after the treatment plan and the orthodontic/surgical procedures were explained to all patients. Forty-six patients were then chosen at random to enter this study ( Figure 1). The selected patient group received information sheets, and their written informed consent was obtained.

The following definitions represent the inclusion criteria: (1) patients aged 18 to 28; (2) unilateral palatally or mid-alveolar upper impaction; (3) distally placement of the impacted canine crown to the lateral incisor root midline; (4) no use of any medications that may affect the orthodontic movement. The following conditions were excluded: (1) bilateral or buccal canine impaction cases; (2) more than 45-degree angle between the canine’s longitudinal axis and the vertical facial plane; (3) presence of lateral incisors root resorption; (4) presence of contact between the canine crown and the lateral incisor root; (5) previous orthodontic treatment; (6) any medical condition that prevents oral surgery; (7) oral structural abnormality that is inherited or congenital.

### Blinding, randomization, and allocation concealment

The participant patients were randomly assigned to the two study groups based on a list of random numbers created using Minitab ^®^ software version 17 (Minitab Inc., State College, Pennsylvania, USA), with a 1:1 allocation ratio. With the use of this randomization process, patients were distributed to the two study groups in an equal numbers: the traditional traction group (TT), which consisted of 23 patients, and the corticotomy-assisted traction group (CAT), which also consisted of 23 patients. Using sequentially numbered, opaque, sealed envelopes, a researcher from the Orthodontic Department who was not involved in this study concealed the allocation.

The principal researcher (MRM) and the patients could not have been blinded during the surgical interventions. Blinding, however, was only achievable during the data analysis stage. In other words, the outcome assessors were blinded and unable to determine to which group the patients belonged.

### Orthodontic procedures

**Figure 2.**
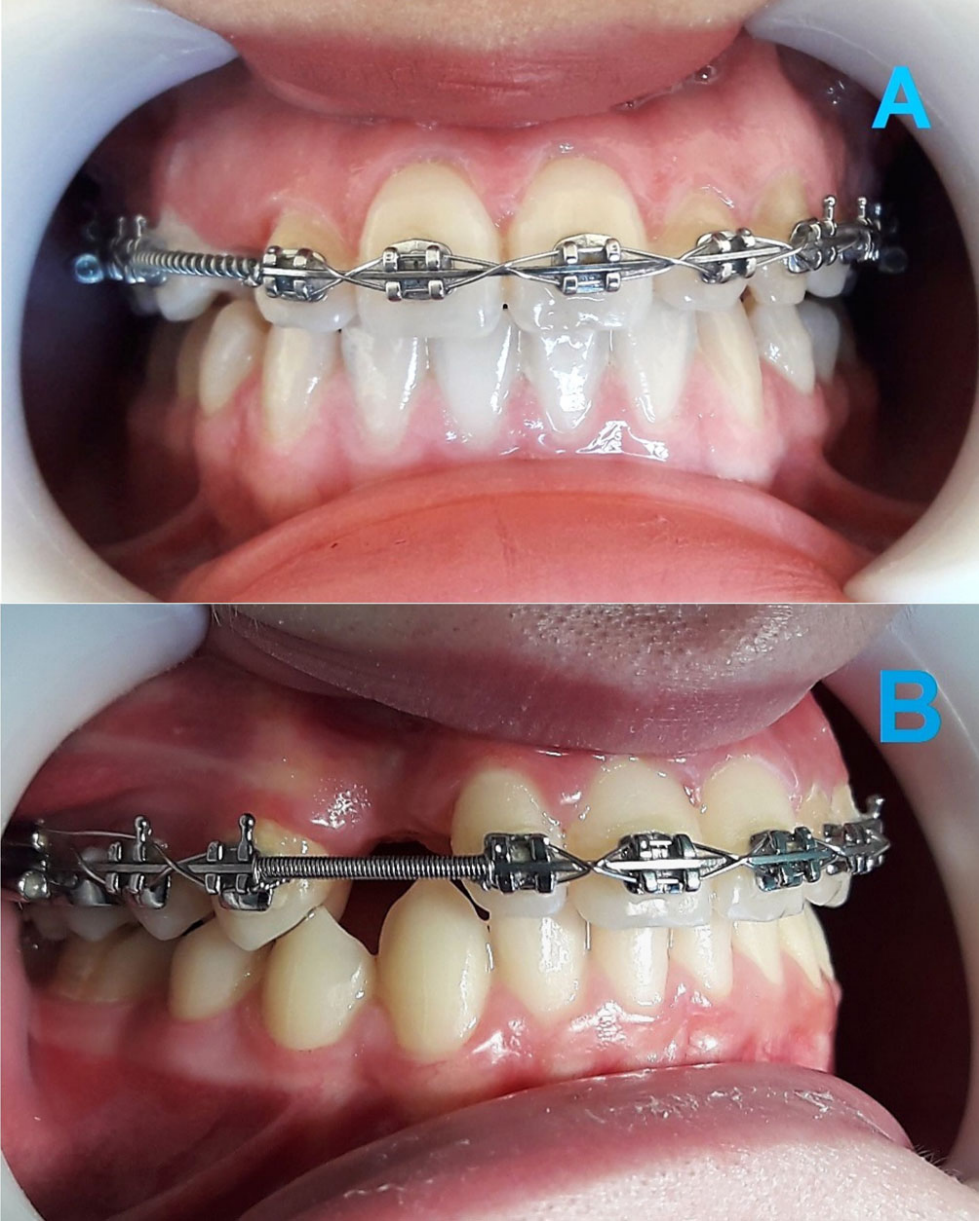
(A) Opening additional space before canine traction using a nickel-titanium open-coil spring. (B) Maintaining the opened space using a stainless steel closed-coil spring.

The aligning and leveling of the maxillary dental arch were performed using the fixed orthodontic appliance (MBT prescription; 0.022-in slot, Votion™, Ortho Technology ^®^, Florida, USA) in both study groups. The following wire sequence was used: (1) 0.012"- or 0.014" nickel-titanium (NiTi); (2) 0.016 × 0.022" NiTi; (3) 0.017 × 0.025" NiTi (NT3 ^®^ SE NiTi Wire, American Orthodontics, Sheboygan, USA); (4) 0.019 × 0.025" stainless steel (American Orthodontics, Sheboygan, WI, USA). This preparatory stage aimed to open sufficient space for aligning the impacted canine within the dental arch. A NiTi open-coil spring (made of 0.010" wire with an inner diameter of 0.030", American Orthodontics, Sheboygan, WI, USA) was used to gain additional space between the lateral incisor and the first premolar, if that distance was not sufficient to accommodate the impacted canine ( Figure 2A). Finally, in order to maintain the opened distance, a stainless steel closed-coil spring (made of 0.010" wire with an inner diameter of 0.030", American Orthodontics, Sheboygan, WI, USA) was inserted ( Figure 2B).

### Surgical procedures

In both study groups, the impacted canines were surgically exposed using the closed flap approaches. Under the direction of one of the co-authors (OH), the same surgeon from the Department of Oral and Maxillofacial Surgery at Damascus University, Faculty of Dentistry, carried out all surgical procedures.

***Traditional traction group (TT)***

**Figure 3.**
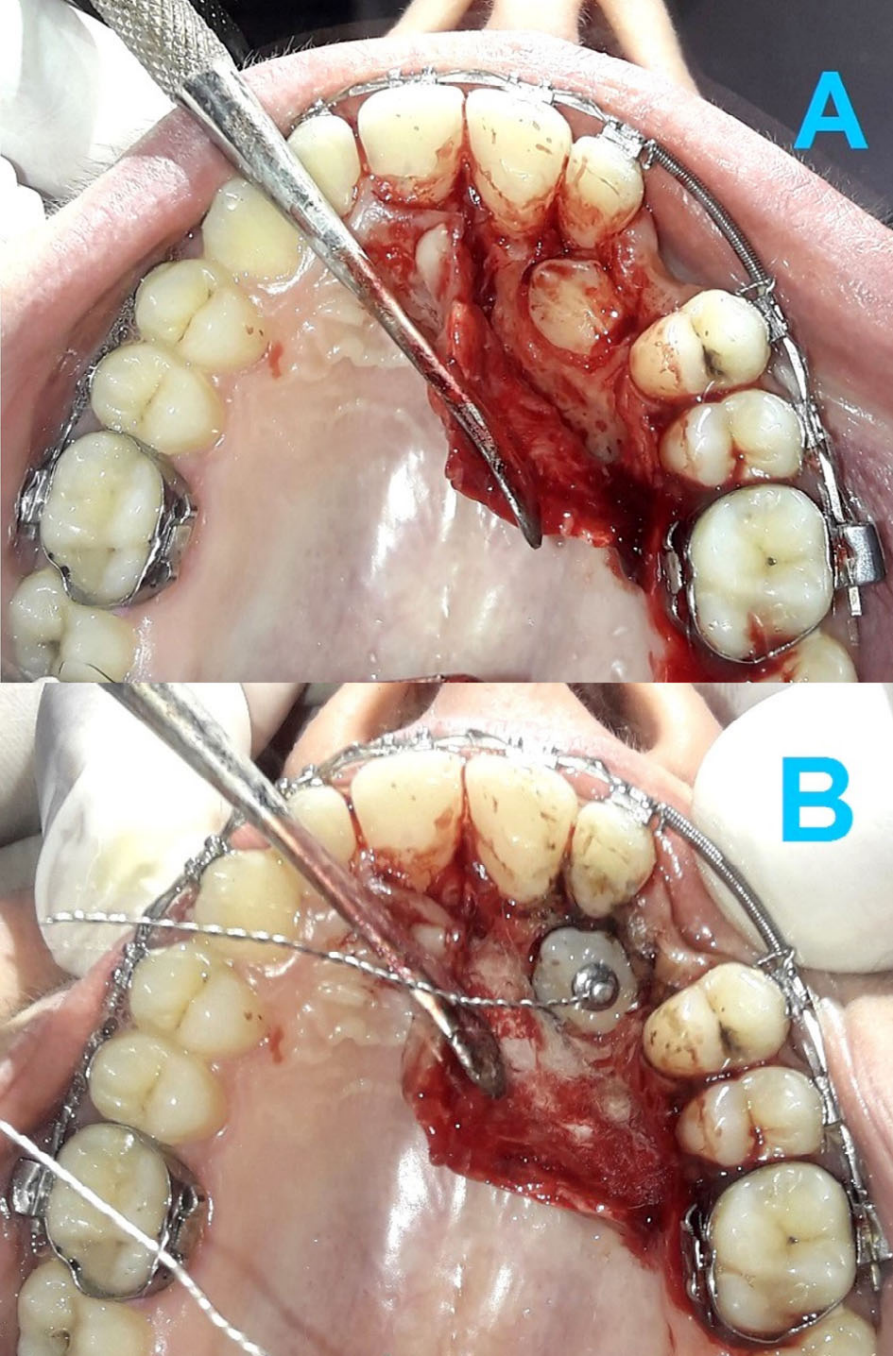
(A) Raising of a full-thickness palatal flap. (B) Bonding an eyelet attachment with a hand-made twisted ligature wire to the exposed canine crown.

Under local anaesthesia (lidocaine HCL 2% - epinephrine 1:80000), a full-thickness palatal flap was raised ( Figure 3A) and the bone overlying the impacted canine crown was removed to reveal a suitable area for attachment bonding, without exposing the cemento-enamel junction (CEJ). An eyelet attachment with a hand-made twisted ligature wire was then bonded to the exposed canine crown ( Figure 3B). The flap was sutured back with the twisted ligature wire extending under the gingival mucosa towards the buccal side. A prescription of an antibiotic (Clavulanic Acid + Amoxicillin, 1000 mg, twice daily for 5 days) and a painkiller (Paracetamol 500 mg, three times daily for 4 days) was given to patients. Patients received guidelines about postoperative oral health care.

***Corticotomy-assisted traction group (CAT)***

**Figure 4.**
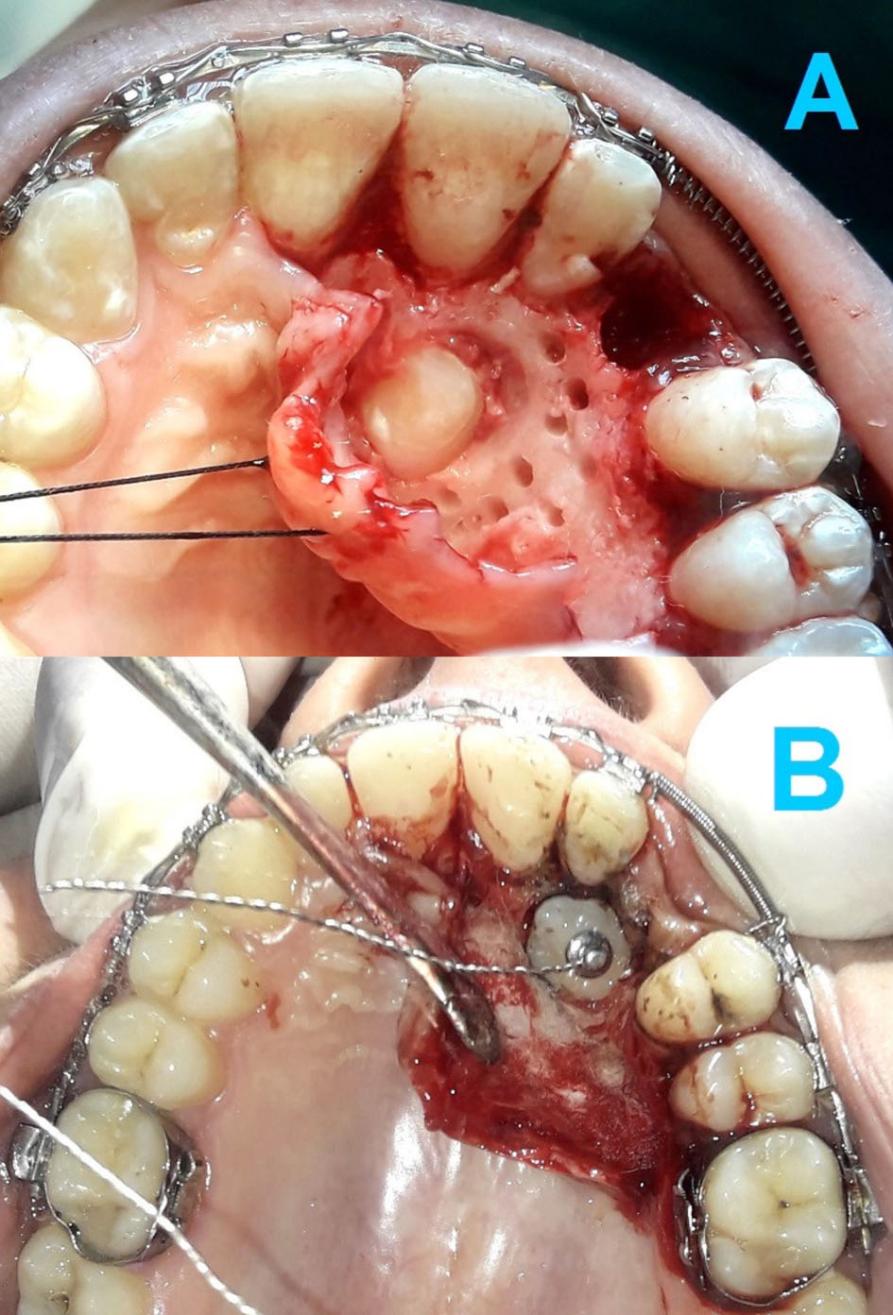
The corticotomy procedures. (A) The first corticotomy procedure involved drilling a series of circular holes (1 mm in diameter, 1-2 mm deep and spaced about 1.5 mm apart) in the cortical bone. (B) The second corticotomy procedure performed using piezosurgery. Two to three vertical gingival incisions with a height of 8 mm were made at the buccal side of the impaction area using a surgical scalpel. Then, the cortical cuts were created using BS1 tip with a cutting depth of 2 to 3 mm and 2-mm spaces between the cuts. Each cut was 1-mm width.

During the surgical exposure phase, patients in this group underwent a corticotomy procedure by drilling a series of circular holes (1 mm in diameter, 1-2 mm deep and spaced about 1.5 mm apart) in the cortical bone. These holes drilled at the area where the impacted canine would be moved towards as well as in the mesial and distal sides of the canine crown ( Figure 4A).

Two months after the first corticotomy intervention, the second flapless corticotomy procedure was performed using piezosurgery (Mectron PIEZOSURGERY ^®^, Carasco, Genova, Italy). Using a surgical scalpel, 2-3 vertical gingival incisions with a height of 8 mm were made during this intervention at the buccal side of the impaction area. Then, the cortical cuts were created using BS1 tip with a cutting depth of 2-3 mm, a 2 mm space between cuts, and a 1 mm cut width ( Figure 4B).

### Follow-up orthodontic procedures

The traction force was applied for the first time two weeks after the surgical exposure using active power chain tied on one end to the twisted ligature wire and on the other end to the first molar band hook on the impaction side. The intensity of the applied traction force was 60 g ^29^ measured using intraoral force gauge (Dentaurum, Ispringen, Germany). Re-activation was done every 2 weeks ^31^ until the appearance of half of the clinical crown of the impacted canine; this moment was considered as the end of the active traction phase. Then, the canine bracket was bonded to start its alignment and levelling stage. Once this stage was complete, the fixed orthodontic appliance was removed, and a vacuum-formed retainer was placed.

### Outcome measures

For all patients, two cone-beam computed tomography (CBCT) images of the maxillary jaw were obtained. The first CBCT image was obtained two weeks before the beginning of treatment while the second image was obtained right on the day the fixed appliance was removed. The CBCT system was a CBCT Picasso ^®^ Pro (Vatech, Seoul, South Korea) with a voxel size of 0.25 mm (FOV 70 × 120 mm), tube voltage of 70 kV, current of 6 mA, and an exposure time of 10 s.

The principal researcher (MRM) performed the measurements and evaluated all variables. The primary outcomes were (1) traction duration, (2) total treatment duration, and (3) the velocity of traction movement. The traction duration is measured as the interval between the onset of orthodontic traction on the impacted canine and the emergence of half of its clinical crown. The total treatment duration calculated as the time between the bonding of the fixed orthodontic appliance until it is removed. The velocity of traction movement is determined by dividing the depth of impaction, which is defined as the distance from the impacted canine cusp tip to the occlusal plane, by the traction duration.

The secondary outcomes were (1) bone support of the aligned canine (BS-IC), (2) bone support of the contralateral naturally erupting canine (BS-CON), (3) bone support of the adjacent lateral incisor (BS-LI), (4) bone support of the adjacent first premolar (BS-PR), and (5) root resorption of the adjacent lateral incisor (RR-LI). Bone support was evaluated on the posttreatment CBCT scans. It was assessed on the mesial, distal, buccal, and palatal aspects of the canines, lateral incisors and first premolar. The total bone support ratio was calculated by averaging the four measurements made on each tooth. The alveolar bone level was measured from the root apex of the evaluated tooth to the alveolar crest, whereas the root length of the evaluated tooth was measured from root apex to the midpoint of a line connecting the mesial and distal points on the cemento-enamel junction of this tooth. The ratio of alveolar bone level to the root length was considered the percentage of bone support. ^32^ The amount of root resorption of the adjacent lateral incisor was evaluated by comparing the root length measured in millimetres before and after treatment.

### The error of the method and the reliability assessment

The method error was assessed by repeating the CBCT measurements for twenty patients selected by random (ten from each group) thirty days after the first measurement. The interclass correlation coefficients (ICCs) were used to determine the presence of any random error, and the paired sample t-tests were used to determine the presence of any systematic error.

### Statistical analysis

Minitab ^®^ statistical package (version 17, State College, Pennsylvania, USA) was used to analyse data. Using Anderson-Darling normality tests, normal distributions were confirmed. Therefore, the two-sample t-test was employed for inter-group comparisons, while the paired sample t-test was used for intra-group comparisons. The two-sample t-tests were used to detect the homogeneity between the two study groups at baseline in terms of age, impaction depth, mesio-distal and buccal-palatal inclinations of the impacted canine longitudinal axis, while the chi-square test was used for gender and impaction site.

## Results

### Patient recruitment and baseline sample characteristics

The baseline characteristics of the sample are shown in Table 1. The study sample consisted 46 patients (13 male and 33 female). There were 39 palatally impacted canines and 7 mid-alveolar impacted canines. There were 23 patients in each group, with a mean age of 20.26±2.17 years and 20.39±2.27 years, in TT and CAT group, respectively. The two study groups did not significantly differ in terms of the initial characteristics of the impacted canine’s position, and they were homogeneous concerning the patient’s age and gender.

**Table 1.** Basic sample characteristics at the beginning of the treatment.

Variable	TT group (n=23)	CAT group (n=23)	P value
**Mean age±SD (years)**	20.26±2.17	20.39±2.27	0.843 ^a^
**Gender (proportion)**	5 M (22%)/18 F (78%)	8 M (34%)/15 F (66%)	0.965 ^b^
**Impaction site bucco-palatally (proportion)**	19 P (82%)/4 Mid (18%)	20 P (86%)/3 Mid (14%)	0.654 ^b^
**Impaction depth±SD (mm)**	8.00±2.20	8.46±2.37	0.514 ^a^
**Mesiodistal inclination±SD (degree)**	30.34±11.33	31.00±11.48	0.848 ^a^
**Buccal-palatal inclination±SD (degree)**	28.15±11.22	28.85±12.48	0.308 ^a^

TT: traditional traction group; CAT: corticotomy-assisted traction group; N: number of patients; SD: standard deviation; M: male; F: female; P: palatal impaction; Mid: mid-alveolar impaction.

^a^ Employing two-sample t-test.

^b^ Employing chi-square test.

### Method error

The ICCs were in the 0.989 to 0.996 range, demonstrating the high reliability of the measurement method. The Paired-sample t-tests results showed that there was no significant difference between the two measurements, indicating the minor and insignificant systematic errors ( Table 2).

**Table 2.** The error of method and the intra-observer reliability.

Variable	Systematic error		Random error			
	Mean difference	P-value *	Intraclass correlation coefficient	95% CI for difference		P-value **
				Lower bound	Upper bound	
**Bone support at the aligned canine**	-0.01	0.560	0.989	0.987	0.991	<0.001
**Bone support at the contralateral canine**	0.02	0.874	0.996	0.990	0.998	<0.001
**Bone support at the adjacent lateral incisor**	-0.01	0.531	0.991	0.986	0.993	<0.001
**Bone support at the adjacent first premolar**	0.02	0.216	0.990	0.988	0.993	<0.001
**Root resorption at the adjacent lateral incisor**	-0.02	0.544	0.991	0.988	0.993	<0.001

* Paired t-tests were used to detect significant differences.

** Intraclass correlation coefficients; P<0.05 was considered statistically significant.

### Outcome measures

The mean duration of active traction was 9.68±3.24 months in the TT group and 6.13±1.81 months in the CAT group with a significant difference between the two groups (P=0.015, Table 3). The mean duration of total orthodontic treatment was 19.98±3.55 months in the TT group and 14.23±1.95 months in the CAT group with a significant difference between the two groups (P=0.001, Table 3). The mean velocity of traction movement was 0.70±0.33 mm/month in the TT group, while it was 1.15±0.35 mm/month in the CAT group with a significant difference between the two groups (P=0.027, Table 3). The duration of active traction decreased by 36%, while the duration of total orthodontic treatment decreased by 29% in the CAT group compared with the TT group. The velocity of traction movement was 39.21% greater in the CAT group than the TT group.

**Table 3.** Descriptive statistics of the traction duration, total treatment duration and traction movement velocity as well as the *P* values of significance tests.

Variable	TT group (n=23)			CAT group (n=23)			TT vs. CAT			P value *
	Mean (SD)	Min	Max	Mean (SD)	Min	Max	Mean difference	95% CI for difference		
								Lower bound	Upper bound	
**Traction Duration (in months)**	9.68 (3.24)	4.00	16.50	6.13 (1.81)	5.00	11.50	3.55	0.43	3.65	0.015
**Total Treatment Duration (in month)**	19.98 (3.55)	11.00	23.50	14.23 (1.95)	10.00	16.00	5.75	2.43	5.65	0.001
**Velocity (in mm/month)**	0.70 (0.33)	0.38	1.54	1.15 (0.35)	0.53	2.16	-0.45	-0.42	-0.02	0.027

P<0.05 was considered statistically significant; TT: traditional traction group; CAT: corticotomy-assisted traction group; N: number of patients; SD: standard deviation; Min: minimum; Max: maximum.

* Two-sample t-test was used.

Regarding the bone support ratio, the differences between the aligned and contralateral canines were non-significant in both study groups (P≈1.000; P=0.559, in the TT and CAT group, respectively; Table 4). No significant differences were found between the two groups when comparing the posttreatment bone support ratios of the aligned canines (P=0.512, Table 5), the adjacent lateral incisors (P=0.379, Table 5), and the adjacent first premolars (P=0.622, Table 5).

**Table 4.** Descriptive statistics of the bone support of the impacted and contralateral canines as well as the *P* values of significance tests.

Group	Impacted canine			Contralateral canine			Impacted vs. Contralateral			P value *
	Mean (SD)	Min	Max	Mean (SD)	Min	Max	Mean difference	95% CI for difference		
								Lower bound	Upper bound	
**TT group**	0.89 (0.04)	0.77	0.94	0.90 (0.03)	0.83	0.95	-0.01	-0.01	0.01	1.000
**CAT group**	0.88 (0.03)	0.85	0.92	0.89 (0.02)	0.86	0.94	-0.01	-0.01	0.02	0.559

TT: traditional traction group; CAT: corticotomy-assisted traction group; SD: standard deviation; Min: minimum; Max: maximum.

* Paired t-test was used to detect the statistically significant differences. P<0.05 was considered statistically significant;

**Table 5.** Descriptive statistics of the bone support of the impacted canine and the adjacent teeth and of the lateral incisor root resorption as well as the P-values of significance tests.

Variable	TT group (n=23)			CAT group (n=23)			TT vs. CAT			P value *
	Mean (SD)	Min	Max	Mean (SD)	Min	Max	Mean difference	95% CI for difference		
								Lower bound	Upper bound	
**Bone support of the aligned impacted canine**	0.89 (0.04)	0.77	0.94	0.88 (0.03)	0.85	0.92	0.01	-0.02	0.01	0.512
**Bone support of the adjacent lateral incisor**	0.85 (0.03)	0.76	0.90	0.86 (0.03)	0.82	0.93	-0.01	-0.04	0.01	0.379
**Bone support of the adjacent first premolar**	0.87 (0.04)	0.81	0.94	0.87 (0.03)	0.81	0.92	0.00	-0.02	0.04	0.622
**Root resorption of the adjacent lateral incisor**	1.22 (1.02)	0.78	1.50	1.30 (1.18)	1.00	1.60	-0.08	-0.90	0.50	0.612
**Number of cases with root resorption (percentage)**	4 (17%)			5 (22%)						

TT: traditional traction group; CAT: corticotomy-assisted traction group; N: number of patients; SD: standard deviation; Min: minimum; Max: maximum.

* Two-sample t-test was used. P<0.05 was considered statistically significant.

The mean bone support ratio of the aligned canines in the TT group was 89% (SD: 0.04), while it was 88% (SD: 0.03) in the CAT group. As for the mean bone support ratio of the contralateral canines, the mean bone support ratio was 90% (SD: 0.03) and 89% (SD: 0.02), in the TT and the CAT group, respectively ( Table 4). The posttreatment values of the bone support ratio of the adjacent laterals was 85% (SD: 0.03), in the TT group and 86% (SD: 0.03), in the CAT group, while it was 87% (SD: 0.04) and 87% (SD: 0.03) for the adjacent first premolars in the TT and the CAT group, respectively ( Table 5).

The mean amount of root resorption of the adjacent laterals was 1.22±1.02 mm in the TT group and 1.30±1.18 mm in the CAT group, with no significant differences between the two study groups (P=0.612, Table 5).

## Discussion

There is little published data on the effect of the corticotomy techniques on the acceleration of palatally impacted canine traction movement, even though these techniques have been shown to be effective in accelerating several orthodontic movements. ^33^^,^^34^ With the exception of the preliminary study conducted by Fischer, ^29^ this randomized controlled trial appears to be the first study that evaluates the efficacy of minimally-invasive corticotomy-assisted orthodontic treatment in accelerating the traction movement of palatally impacted maxillary canines and the associated dento-alveolar changes compared with the traditional surgical/orthodontic treatment. The homogeneity of the patient sample and the initial positioning of the impacted canines enabled such a comparison.

Our study findings showed that both the duration of active traction and the total treatment duration were significantly shorter in the CAT group compared to the TT group. In other words, the velocity of traction movement in the CAT group was significantly greater. This can be attributed to the fact that the corticotomy interventions decreased the cortical bone mass and its resistance to the impacted canine forced eruption movement due to its weakening by cortical cuts. ^29^ Furthermore, the stimulation of the regional acceleration phenomenon (RAP) caused by corticotomy interventions may have stimulated the expression of inflammatory markers and cytokines which led to increased osteoclast activity, stimulated bone remodelling and reinforced blood circulation in the alveolar bone, which led to acceleration of the orthodontic traction movement. ^35^^,^^36^

The results of the current study were in line with the previous studies that compared the acceleration and the conventional traction methods in terms of the incidence of acceleration but were inconsistent with those studies’ results regarding the amount of acceleration obtained. The mean traction movement velocity in the current study was greater (0.37 mm/wk.) than that of Fischer (0.26 mm/wk.). ^29^ This difference might be attributed to the corticotomy procedure performed twice from both the buccal and palatal sides in the current study, which further weakened the resistance of the cortical bone to the forced eruption movement, In addition to the occurrence of additional stimulation of the RAP.

In contrast, Ferguson et al. ^30^ reported a larger movement acceleration ratio (about 65%) than the current study (about 36%). This can be explained by the difference in the acceleration technique used, the mean ages of the participants and the removal of a greater amount of bone surrounding the impacted canine in that study compared to the current study. Moreover, Ferguson *et al*. ^30^ included cases of unilateral and bilateral PICs and both surgical exposure approaches (open and closed traction methods) were used in that study. Although the results of the current study were consistent with those of Yussif *et al*., ^31^ a direct comparison with that study was not possible due to the difference in the acceleration method that was used, i.e., local injections of vitamin C.

This study showed that the posttreatment bone support ratios of the aligned canines did not differ significantly between the TT and CAT groups as well as when compared to the contralateral canines in both study groups. In addition, the results of the inter-group comparison showed that there were no significant differences in the mean bone support ratios of the adjacent lateral incisors and the first premolars. These results can be explained by the use of the same orthodontic traction techniques in both study groups, the use of light traction force in a direction that resembles the normal eruption path, and the fact that the amount of bone removed during surgical interventions was minimal. The results of the current study were in agreement with those of Fischer ^29^ who found a non-significant difference in bone level of the PICs treated by the corticotomy-assisted withdrawal method compared to the traditional traction method.

No clinical trials have been published in the medical literature evaluating the level of bone support of the teeth adjacent to the PICs underwent corticotomy-assisted orthodontic treatment until now. Therefore, the results of the CAT group in the current work cannot be compared with others due to the lack of similar studies.

The mean amount of root resorption of the adjacent laterals was almost similar in the two study groups with no significant differences. The light traction force used and its direction that from the beginning of its application was away from the root of the lateral incisors, could explain the presence of the low percentage and mild degree of root resorption of the adjacent laterals. Due to the lack of similar studies, the results obtained in the current study are only compared with the results of studies that used the traditional traction methods.

The results of the current study were consistent with those of Arriola-Guillén *et al*., ^37^ who found that the amount of root resorption of adjacent laterals did not exceed 2 mm after the treatment of unilateral vs. bilateral palatally impacted canine cases by the closed traction method. Our results were also in agreement with those of Lempesi *et al*. ^38^ who evaluated the root resorption of maxillary incisors induced by the orthodontic treatment for patients with upper impacted canines and found a range of 0.86-1.17 mm of root resorption of adjacent laterals.

### Limitations

The current study included a specific type of upper canine impactions. Therefore, the effects of different probabilities of the initial location, mesiodistal and bucco-lingual angulation of the impacted canines on the traction movement velocity were not evaluated. Secondly, only one type of mechanical traction means was used and the effectiveness of other traction techniques were not evaluated. Thirdly, this trial did not assess the effects of gender and age on the traction movement rate, for which a larger sample size would be necessary.

### Generalizability

As the present study included particular impaction conditions with particular impacted canine’s locations and conducted on a specific patient group treated with specific mechanical orthodontic and surgical techniques in only one teaching hospital, the results of the current study can be limitedly generalized. In order to acquire results that are more generalizable, more randomized controlled trials with larger sample sizes should be carried out in diverse populations with various types of canine impaction.

## Conclusions

Orthodontic traction assisted with minimally-invasive corticotomy was effective in increasing the rate of traction movement of the palatally impacted canines, thereby decreasing the traction duration and the overall treatment duration. The acceleration surgical interventions used did not cause significant changes in the bone support ratios of the aligned canines and the adjacent teeth compared with the traditional traction method. Finally, the acceleration procedure did not result in any significant difference regarding the amount of root resorption of adjacent laterals compared with the traditional treatment methods.

## Ethical statement

The approval of the Local Research Ethics Committee of Damascus University was obtaining before conducting this study (UDDS-499-01022020/SRC-2195). This study was registered at ClinicalTrials.gov and was funded by the Postgraduate Research Budget of Damascus University (Ref no: 83005751015DEN). The selected patient group received information sheets, and their written informed consent was obtained.

## Data Availability

Figshare. Accelerated Traction of Impacted Canines. DOI: https://doi.org/10.6084/m9.figshare.22926152.v1. ^39^ This project contains the following underlying data:
• 01 Baseline Sample Characteristics.xlsx. (This file contains the data used to summarize the characteristics of the include patients in this study). • 02 Duration of treatment.xlsx (This file contains the variables used to measure the duration of the provided treatment for each patient). • 03 Bone Support and Root Resorption.xlsx (This file contains the variables used to evaluate the percentages of bone support and the amount of root resorption observed on the lateral incisors adjacent to the impacted canines) • Information Sheet.pdf (This is the information sheet distributed to the candidate patients for inclusion in this study) • CONSORT2010 checklist v01.pdf (This checklist contains the 25 items of the CONSORT guidelines and the pages where these items can be found). Data are available under the terms of the Creative Commons Zero “No rights reserved” data waiver (CC0 1.0 Public domain dedication).
