# Supplementary material for: The effectiveness of minimally-invasive corticotomy-assisted orthodontic treatment of palatally impacted canines compared to the traditional traction method in terms of treatment duration, velocity of traction movement and the associated dentoalveolar changes: A randomized controlled trial
Source: F1000Res. 2023 Jun 19;12:699. [Version 1] doi: 10.12688/f1000research.135338.1 (PMC10618642; doi:10.12688/f1000research.135338.1)
Supplement: Peer review history

# Reviewer report: Reviewer response for version 1

**DOI:** 10.5256/f1000research.148452.r215289

**Published:** 2023-12-15

**Authors:** Huang Li

**Affiliations:** 1 Nanjing University, Nanjing, China

The study was to evaluate the effectiveness of a minimally-invasive corticotomy-assisted treatment of palatally impacted canines (PICs) compared with the traditional method by evaluating treatment time, the velocity of movement, and the associated dentoalveolar changes. It was an interesting study, but still needs to address the following questions-

 1. For palatally impacted canines, the traditional treatment during surgical procedure was to remove some bone and reduce some obstacle. Therefore, the corticotomy seems not very necessary from this point.

 2. The duration time was closely related to impaction distance. it is very important to analyze the detailed result.

 3. The literature showed  "They found that the duration of treatment was 25.8 months for unilateral impaction cases, and in bilateral cases, the duration of treatment was 32.3 months." Actually, the treatment time in this study was too short compared to the previous study.

 4. The author should simplify the introduction, not like a review to show all studies.

 5. Figure 3B and Figure 4B look like the same picture. Please clarify it.

Is the work clearly and accurately presented and does it cite the current literature?

Partly

If applicable, is the statistical analysis and its interpretation appropriate?

I cannot comment. A qualified statistician is required.

Are all the source data underlying the results available to ensure full reproducibility?

Yes

Is the study design appropriate and is the work technically sound?

Partly

Are the conclusions drawn adequately supported by the results?

Yes

Are sufficient details of methods and analysis provided to allow replication by others?

Partly

Reviewer Expertise:

orthodontics

I confirm that I have read this submission and believe that I have an appropriate level of expertise to confirm that it is of an acceptable scientific standard, however I have significant reservations, as outlined above.

# Reviewer report: Reviewer response for version 1

**DOI:** 10.5256/f1000research.148452.r179862

**Published:** 2023-10-31

**Authors:** Ashraf Ayoub

**Affiliations:** 1 Scottish Craniofacial Research Group, Glasgow University Dental Hospital and School, School of Medicine, College of Medical, Veterinary and Life Sciences, University of Glasgow, Glasgow, UK

This is an excellent piece of research which is based on randomized control trial at the top of the hierarchy of scientific evidence in the literature. The study was well conducted, the results are comprehensive and answers the research question. I have recommended the indexing of the manuscript, with minor corrections, in recognition of its high quality. The following minor corrections may be considered:

1. I suggest the "velocity" of tooth movement should be replaced by the the "average rate".
2. What is (BS1)?
3. A new photo (4B) showing the buccal gingival cuts and the cortctotomy would be ideal.
4. Please add a sentence regarding the hypothesis that was tested in this study.
5. Please add a sentence regarding future studies.

Congratulations for this excellent piece of research.

Is the work clearly and accurately presented and does it cite the current literature?

Yes

If applicable, is the statistical analysis and its interpretation appropriate?

Yes

Are all the source data underlying the results available to ensure full reproducibility?

Yes

Is the study design appropriate and is the work technically sound?

Yes

Are the conclusions drawn adequately supported by the results?

Yes

Are sufficient details of methods and analysis provided to allow replication by others?

Yes

Reviewer Expertise:

Orthognathic surgery, 3D image analysis, bone bioengineering.

I confirm that I have read this submission and believe that I have an appropriate level of expertise to confirm that it is of an acceptable scientific standard.

# Reviewer report: Reviewer response for version 1

**DOI:** 10.5256/f1000research.148452.r179863

**Published:** 2023-07-18

**Authors:** Iyad K. Al-Omari

**Affiliations:** 1 Department of Orthodontics and Pediatric Dentistry, School of Dentistry, The University of Jordan, Amman, Jordan

The effectiveness of minimally-invasive corticotomy-assisted orthodontic treatment of palatally impacted canines compared to the traditional traction method in terms of treatment duration, velocity of traction movement and the associated dentoalveolar changes: A randomized controlled trial.

 This is an interesting study that is investigating the effectiveness of minimally-invasive corticotomy-assisted orthodontic treatment of palatally impacted canines compared to the traditional traction method. The RCT design of this manuscript as well as the layout is nicely written. There are, however, few minor modifications that need to be addressed by the authors. My recommendation is to accept this manuscript.

 Title:

 The title is too long, please consider rewriting the title to indicate the main key factors that are investigated in this study, it could be shortened as follows: The effectiveness of minimally-invasive corticotomy-assisted orthodontic treatment of palatally impacted canines compared to the traditional traction method: A randomized controlled trial.

 Introduction:

 The literature review is too long and the authors provide a detailed review of several similar key studies (page 2: 2 ^nd^, 5 ^th^ and 9 ^th^  paragraphs). Please consider critically reviewing these studies in a summarized concise manner and highlighting any shortcoming of these studies.  

 Consider replacing the word “troubling” with “challenging” in the text: “The time required for orthodontic traction of impacted canines is a particularly troubling clinical problem because it prolongs the orthodontic treatment duration.”

 Methods:

 The authors need to justify and discuss why they considered the use of a closed surgical technique to align the palatally impacted canines. The literature supports the use of open surgical technique to align impacted canines that are palatally positioned.

 Results:

 This section could be modified in order to avoid repeating the reported results in different format. The authors could describe the general trends of the results in text  and refer the reader to tables and figures instead of reporting the same numbers, values and percentages in text and tables.

 Figures and Tables:

 Figure 1 and 2: please consider removing these figures as it does not illustrate any details relevant to the investigated variables

 Figure 4B: there is a confusion with regards to the description of procedure on the buccal side of the impaction while the photographs depict a palatal view of the impaction. Please consider replacing the photograph with an appropriate illustration of the procedure performed in the buccal side of the impaction.

 Table 2: no need to include this table as the results are reported in full in text. Please consider removing this table    

    

Is the work clearly and accurately presented and does it cite the current literature?

Yes

If applicable, is the statistical analysis and its interpretation appropriate?

Yes

Are all the source data underlying the results available to ensure full reproducibility?

Yes

Is the study design appropriate and is the work technically sound?

Yes

Are the conclusions drawn adequately supported by the results?

Yes

Are sufficient details of methods and analysis provided to allow replication by others?

Yes

Reviewer Expertise:

Orthodontic treatment, cleft lip and palate, Oral health ralted quality of life

I confirm that I have read this submission and believe that I have an appropriate level of expertise to confirm that it is of an acceptable scientific standard.
